# Unveiling the structural features that determine the dual methyltransferase activities of *Streptococcus pneumoniae* RlmCD

**DOI:** 10.1371/journal.ppat.1007379

**Published:** 2018-11-02

**Authors:** Yiyang Jiang, Hailong Yu, Fudong Li, Lin Cheng, Lingru Zhu, Yunyu Shi, Qingguo Gong

**Affiliations:** Hefei National Laboratory for Physical Science at the Microscale, School of Life Sciences, University of Science and Technology of China, Hefei, Anhui, China; National Jewish Health, UNITED STATES

## Abstract

Methyltransferase RlmCD was previously shown to be responsible for the introduction of C5 methylation at both U747 and U1939 of the 23S ribosomal RNA in *Streptococcus pneumoniae*. Intriguingly, its structural homologue, RumA, can only catalyze the methylation of U1939, while RlmC is the dedicated enzyme for m^5^U747 in *Escherichia coli*. In this study, we describe the structure of RlmCD in complex with its cofactor and the RNA substrate containing U747 at 2.00 Å or U1939 at 3.10 Å. We demonstrate that multiple structural features collaborate to establish the dual enzymatic activities of RlmCD. Of them, the side-chain rearrangement of F145 was observed to be an unusual mechanism through which RlmCD can discriminate between U747- and U1939-containing RNA substrate by switching the intermolecular aromatic stacking between protein and RNA on/off. An in-vitro methyltransferase assay and electrophoretic mobility shift assay were performed to validate these findings. Overall, our complex structures allow for a better understanding of the dual-functional mechanism of RlmCD, suggesting useful implications for the evolution of the RumA-type enzyme and the potential development of antibiotic drugs against *S*. *pneumoniae*.

## Author summary

Methylation of nucleotides widely occurs on ribosomal RNAs (rRNAs) in all living organisms; some clusters at the functional centers of rRNA are important for protein translation. In pathogenic bacteria, it has been shown that modulation of the methylation state of some rRNA nucleotides serves as a clinically important mechanism to confer resistance to ribosome-targeting antibiotics. Because some methyltransferases can introduce methylation at different target sites, understanding the functional roles of each individual methylation site in rRNA has become meaningful but remains challenging. In this research, using protein-RNA crystal structures, we demonstrate that multiple structural determinants collaborate to establish the dual enzymatic activities of RlmCD, which has been previously shown to catalyze C5 methylation at both U747 and U1939 of 23S rRNA in some gram-positive bacteria. This structural biology study provides a new perspective for us to understand the dual-functional mechanism of RlmCD, suggesting useful implications for the evolution of RumA-type enzyme and the potential development of antibiotic drugs against *Streptococcus pneumoniae*.

## Introduction

RNA methylation is an abundant post-transcriptional modification occurring in almost all types of RNA molecules from three kingdoms of life. The introduction of methylation into RNAs is conducted by RNA methylation enzymes (methyltransferase, MTase) with diverse catalytic mechanisms [1, 2]. As the key component of protein synthesis machinery in all living organisms, ribosomal RNA (rRNA) is the one of the RNA molecules with most methylation and other types of modifications. For instance, 10 and 14 methylation have already been identified in the 16S and 23S rRNA of *E*. *coli*, respectively, with a variety of methylation types including 1-methylguanosine (m^1^G), 3-methyluridine (m^3^U), 5-methyluridine (m^5^U), 5-methylcytidine (m^5^C) and 2-methyladenosine (m^2^A) [3, 4]. Although the majority of these methylations is clustered at the functionally important sites of rRNA, such as the peptidyl transferase center (PTC), the nascent peptide exit tunnel (NPET), and the A, P, and E sites of tRNA binding sites, none of them has been shown to be critical for cell survival in prokaryotes [5]. However, recent findings suggest that rRNA modifications may serve as an important source in prompting ribosome heterogeneity related to the certain function of ribosome in response to environmental stress [6]. On the other hand, with the wide use of antibiotic drugs targeting the bacterial ribosome, modulation of rRNA methylation has emerged as a common mechanism of antibiotic resistance or susceptibility [7]. Aberrant methylation (either hypermethylation or loss-of-methylation) serves as an important way to antagonize the function of antibiotics under certain circumstances [8–10].

One highly modified site in bacterial rRNA, which is in close proximity to NPET, is constituted by the central loop in domain V and the loop of hairpin 35 in domain II of 23S rRNA [11]. A family of structurally similar macrolide and ketolide antibiotics bind at this site to stop bacterial growth by interfering with protein synthesis [7, 8, 12]. Previous researches have revealed multiple modified nucleotides at this site, including m^1^G745, Ψ746, m^5^U747, m^1^G748 from domain II and m^6^A2058 from domain V [13, 14]. In addition, m^1^G745 and m^1^G748 are mutually exclusive modifications occurring in gram-negative and gram-positive bacteria, respectively [15]. Much evidence has already indicated that different methylation status relating to these nucleotides are intimately correlated with the resistance or susceptibility of some antibiotic drugs, such as erythromycin (ERY), telithromycin (TEL), and solithromycin (SOL) [9, 16–18]. For instance, in gram-positive bacteria *S*. *pneumoniae*, dimethylation of A2058 modified by Erm(B) methyltransferase confers moderate TEL resistance, while m^1^G748 catalyzed by RlmA^II^ further stabilizes the interaction of TEL with domain II of 23S rRNA and therefore the inactivation of RlmA^II^ confers higher-level TEL resistance in the *erm*(B)-carrying strain [19–21]. Moreover, C5 methylation of U747 was recently found to contribute to TEL susceptibility by promoting an efficient RlmA^II^-mediated G748 methylation [22].

As the only two m^5^U-modified rRNA nucleotides in bacteria, U747 and U1939 are located at the loop of hairpin 35 in domain II and at the U1939-containing loop in domain IV, respectively, with a ~ 50 Å distance between them in a mature prokaryotic rRNA [23]. Although it has been reported that U1939 methylation is involved in reproducible resistance to fusidic acid and capreomycin, its working mechanism is less understood compared to m^5^U747 [24]. Methylation at U747 in *E*. *coli* is catalyzed by MTase RlmC, while RumA (or RlmD) is the specific m^5^U MTase for U1939 in gram-negative bacteria, and its catalytic mechanism has been well illustrated [14, 25]. However, in *S*. *pneumoniae* and *Bacillus subtilis*, which both belong to gram-positive bacteria, RlmCD and its homologue YefA have been proven to be dual-functional methyltransferases to catalyze both m^5^U747 and m^5^U1939 in 23S rRNA [22, 26]. A handful of modifying enzymes have the tendency to modify multiple nucleotides that are immediately adjacent or within a range of several nucleotides on the bacterial rRNAs [27, 28]. In very rare cases, including RlmCD and YefA, however, an rRNA enzyme can catalyze modifications at distant locations, implicating that RlmCD and/or YefA may have the capacity to specifically recognize and bind to different sites of ribosomal RNA.

**Fig 1:**
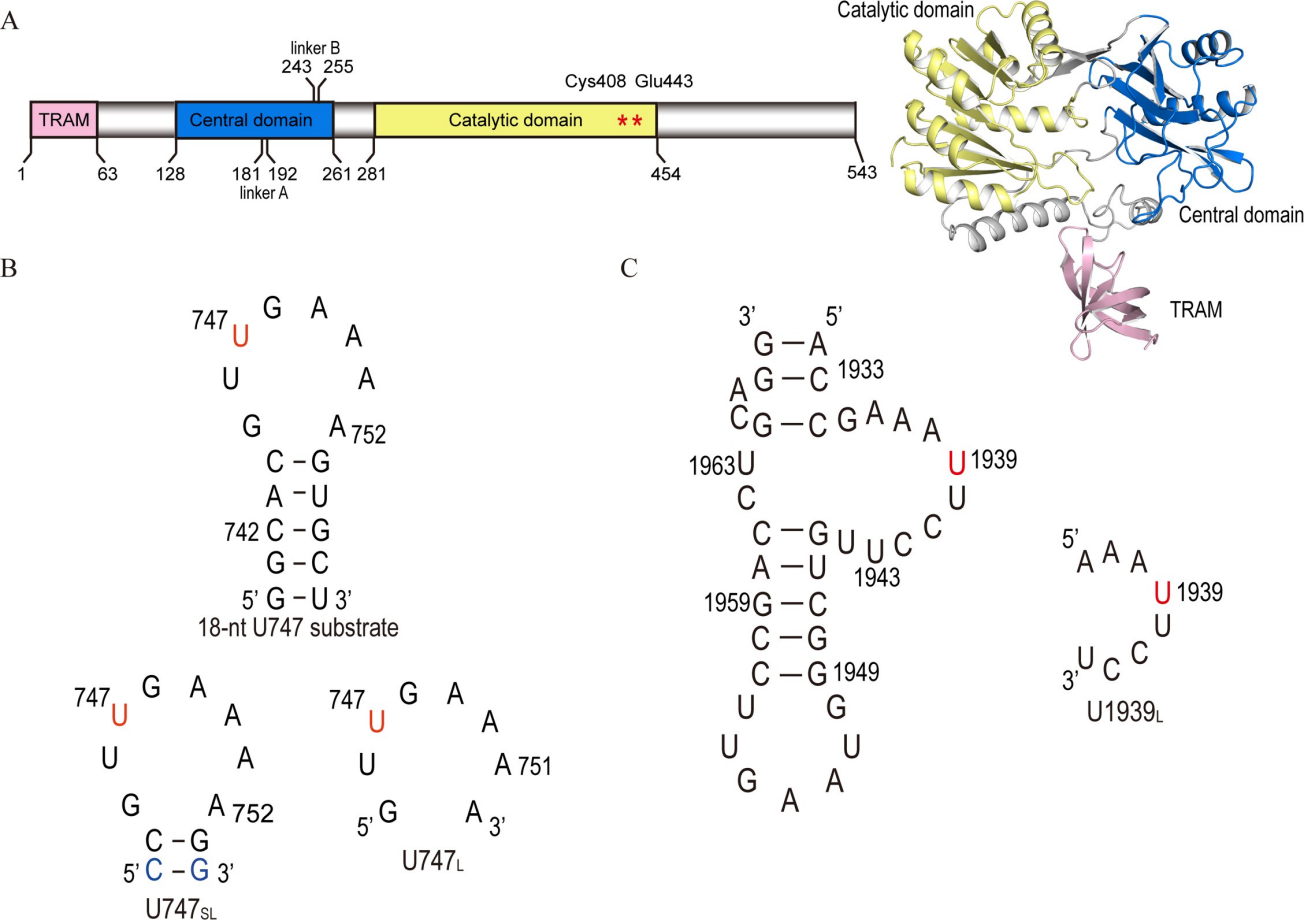
A brief introduction of RlmCD and its RNA substrates. (A) Schematic illustration of the full-length RlmCD. (B, C) Primary sequences and secondary structures of RNA segments analogous to U747 and U1939 sites of *S*. *pneumoniae* 23S rRNA. The nucleotides in red represent the methylation target (U747 or U1939) of RlmCD in the RNA segments while the nucleotides in blue are 743–754 base-pair which were mutated from original A-U to G-C to increase the conformational stability of U747_SL_.

We previously determined the crystal structure of apo-form RlmCD, which highly resembles that of RumA and contains three individual domains, including the N-terminal TRAM domain, central domain, and C-terminal catalytic domain (Fig 1A) [29]. In further attempts to explore the specific recognition of RlmCD for the U747 site of 23S rRNA, however, the complex structure of RlmCD with S-adenosyl homocysteine (SAH) and an 18-nt U747-containing RNA segment that we obtained only reflect a physiologically irrelevant interaction, likely due to crystal packing. The structural basis of RlmCD for its recognition of two distinctive sites on 23S rRNA remains elusive because the structures of RlmCD and RumA are highly similar (RMSD value of 274 atoms C_α_ is 1.9 Å) and RumA can only catalyze the methylation of U1939 in gram-negative bacteria.

To uncover the structural features determining the multi-selectivity of RlmCD for RNA substrates, further work has been conducted for this report to obtain the crystal structures of RlmCD in complex with re-designed U747 RNA substrates and the U1939 RNA substrate. Through careful structural comparison between RlmCD complexed with U747 and U1939 RNA substrates, we identified multiple crucial residues that behave differently in two specific recognitions. Further in-vitro MTase and EMSA assays were employed to confirm our observations from the structures. The structural insights we gained in this research provide a new perspective for us to understand the delicate mechanism that RlmCD employs to recognize different RNA substrates.

## Results

### RNA substrate design and complex structure determination

In a previous attempt to obtain the complex structure of RlmCD and RNA substrate, we used an 18-nt RNA segment (5′-^740^GGCACGUm^5^UGAAAAGUGCC^757^-3′) analogous to the major portion of the U747-methylated 23S rRNA hairpin 35 in *S*. *pneumoniae* (Fig 1B). In the final model we obtained, however, the double-helical region of RNA hairpin was found to bind at the cleft formed between the N-terminal TRAM domain and the catalytic domain, while the electron density corresponding to the loop region was not observed. This interaction, however, was eventually proven to be functionally irrelevant and was probably caused by crystal packing or the strong ability of the cleft to accommodate double-helical RNA [29].

To obtain the complex structure reflecting the actual recognition of RlmCD for the U747 site of 23S rRNA, we re-designed the RNA substrate using a guiding strategy to weaken the interaction between the RNA helical region and RNA-binding cleft of RlmCD. An 8-nt (5′-^745^GUUGAAAA^752^-3′, hereafter termed as U747_L_) or 12-nt (5′-^743^CCGUUGAAAAGG^754^-3′, U747_SL_) U747-containing RNA analogue (Fig 1B) representing the loop region of hairpin 35 alone or with two additional adjacent base pairs (^743^C-G^754^ and ^744^C-G^753^) was therefore crystalized with the construct of 1–454 of RlmCD containing a E443Q mutation, using S-adenosyl methionine (SAM) as the cofactor. The 743–754 base-pair in U747_SL_ was modified to a G-C from an A-U to increase the stability of the RNA structure. A similar RNA design was also applied to co-crystalize the RlmCD with SAM and an 8-nt U1939-containing RNA stretches (5′-^1936^AAAUUCCU^1943^-3′, U1939_L_) (Fig 1C) to understand the mutual recognition between RlmCD and U1939 site on 23S rRNA.

**Fig 2:**
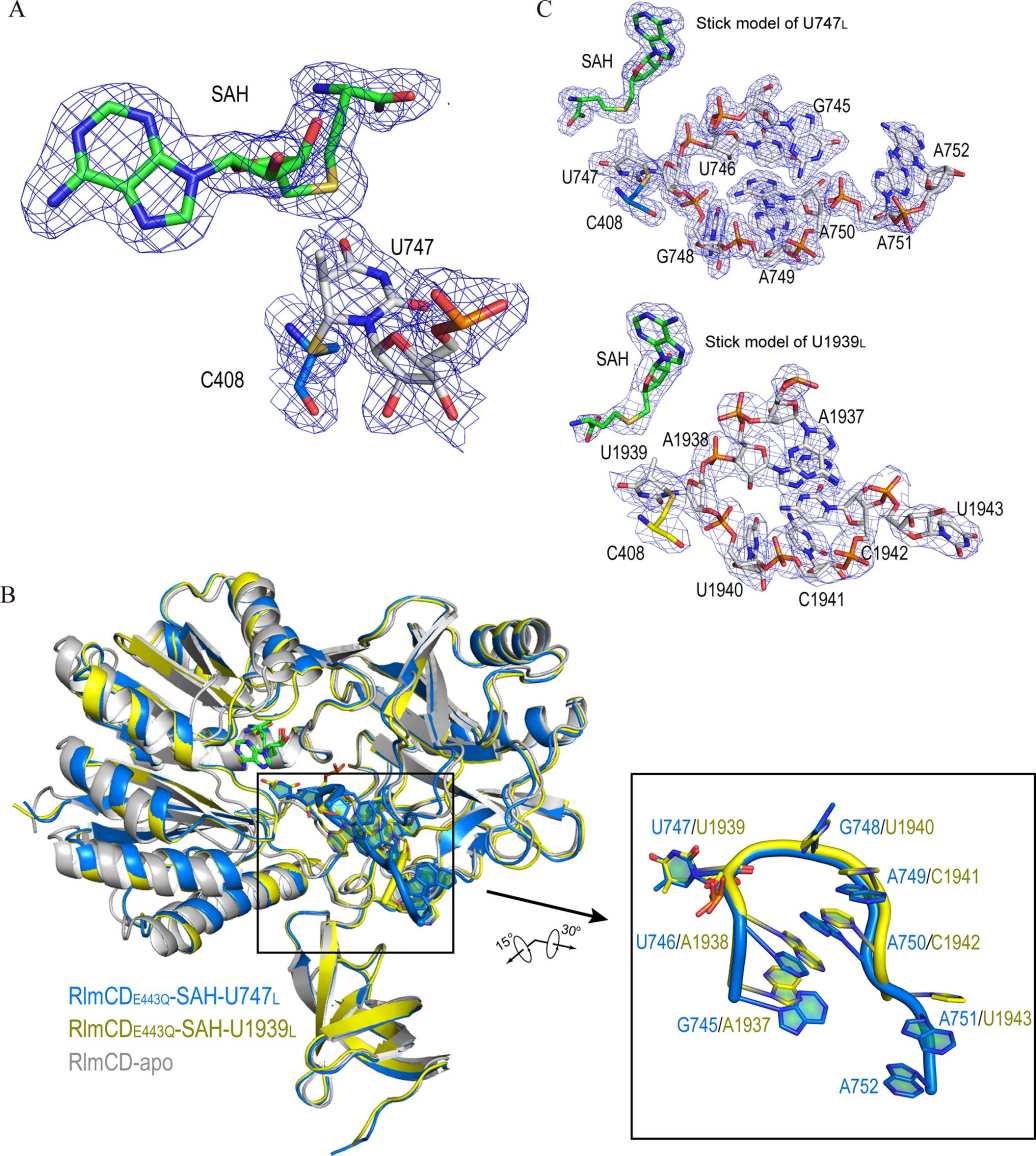
Complex structures of RlmCD with RNA substrates and cofactors represent an intermediate state of methyl transfer reaction. (A) In active site of RlmCD complexed with U747_L_, the cofactor is modeled as SAH and the electron density of the methyl group is observed attaching to the base C5 atom of U747. A covalent bond is formed between the C6 atom of U747 and side-chain sulfur atom of RlmCD C408. (B) Superimposition of apo-form RlmCD with RlmCD-SAH-U747_L_ and RlmCD-SAH-U1939_L_ complex structures (RMSD values for Cα atoms are 1.0 Å and 1.2 Å, respectively). (*Inset*) Close-up of overall structures of U747_L_ and U1939_L_. Structure orientations are slightly adjusted to exhibit a clearer effect. (C) Structures of U747_L_ and U1939_L_ are individually shown in stick model. Electron density map with 2Fo-Fc calculated at 1.0σ shown for SAH, RlmCD C408, and all RNA nucleotides in both (A) and (C).

All crystal structures were solved by molecular replacement using the structure of the apo-form RlmCD (PDB ID 5XJ1) as the search model (Table 1), and the electron densities of all nucleic acids were modeled accordingly [30]. In all structures, the cofactors were modeled as SAH, due to the missing electron density of the methyl group, while its density was clearly observed attaching to the base C5 atom of U747 or U1939 (Fig 2A and S1A and S1B Fig), indicating that the complexes gained in the crystal structures all exist as the trapped enolate intermediate 3 (S1C Fig), given that E443Q eliminates the final step of proton abstraction from C5 [25, 31]. This judgment was further confirmed by the observation that a covalent bond is formed between the C6 atom of U747 or U1939 and side-chain sulfur atom of C408 in all complex structures (Fig 2A and S1A and S1B Fig). In addition, in the RlmCD-SAH-U747_SL_ complex structure crystalized in space group *P*1, each asymmetry unit contains two complex molecules that pack together through RNA-RNA intermolecular base stacking (S2A Fig). All structure determination and refinement statistics were listed in Table 1.

**Table 1:** Data collection and refinement statistics. Values in parentheses are for the highest resolution shell.

Data collection statistics	RlmCD-SAH-U747_L_	RlmCD-SAH-U747_SL_	RlmCD-SAH-U1939_L_	RlmCD-SAH-U1939_L_(3.24Å)
**PDB ID**	5ZQ0	5ZQ8	5ZQ1	5ZTH
**Data collection**				
Wavelength (Å)	0.9778	0.9791	0.9791	0.9774
Space group	*P*2_1_2_1_2_1_	*P*1	*P*2_1_2_1_2_1_	*P*2_1_2_1_2_1_
Cell dimensions				
*a*, *b*, *c* (Å)	47.41,95.81,114.21	62.23,62.26,79.82	47.39,96.10,114.16	47.95,95.53,114.51
*α*, *β*, *γ* (°)	90,90,90	74.58, 85.47,65.03	90,90,90	90,90,90
Resolution range (Å)	50.00–2.00(2.03–2.00)	56.38–2.18(2.23–2.18)	40.00–3.10(3.15–3.10)	50.00–3.24(3.30–3.24)
*R*_merge_ (%)	8.0(47.7)	13.0(55.1)	16.3(84.4)	31.4(86.3)
*I/σI*	24.4(4.1)	6.2(2.2)	13.7(3.2)	5.8(2.0)
*CC*_*1/2*_	0.999(0.952)	0.981(0.828)	0.966(0.801)	0.978(0.808)
Completeness (%)	100.0(100.0)	87.8(95.4)	99.9(100.0)	100.0(100.0)
Multiplicity	11.9(12.1)	3.7(3.7)	8.4(8.6)	9.6 (9.5)
Wilson B-factor (Å^2^)	24.6	35.4	67.8	47.0
**Refinement**				
Number of reflections(over all)	35929	54930	9526	8990
Number of reflections (test set)	1813	2643	513	464
*R*_work_/*R*_free_ (%)	18.9/21.2	25.5/30.3	19.5/23.7	18.4/25.3
Number of atoms				
Protein/Ligands/Water	3623/198/204	6978/570/67	3600/189/0	3608/189/0
*B*-factors (Å^2^)				
Protein/Ligands/Water	26.55/30.65/29.56	41.92/33.45/31.73	65.01/82.62/-	44.36/58.70/-
R.M.S. deviations				
Bond length (Å)	0.005	0.006	0.007	0.007
Bond angles (°)	1.0141	1.129	1.256	1.203
Ramachandran plot (%)				
Favored/Allowed/Outlier (%)	97.1/2.5/0.4	95.0/3.3/1.7	95.1/4.7/0.2	96.2/3.6/0.2

### Overall structures of RlmCD-SAH-U747_L_ and RlmCD-SAH-U1939_L_

The conformations of RlmCD in the complex structures are highly similar to that of apo-form RlmCD (RMSD of C_α_ are 0.997 Å for U747_L_ complex and 0.995 Å for U1939_L_ complex), without obvious relative motion between different domains upon the RNA binding (Fig 2B). In two complex structures of RlmCD with U747_L_ and U747_SL_, the overlapped RNA loop regions show a highly similar U-shape conformation, with the U747 base flipped out of the RNA loop and engaged into the active site in the C-terminal catalytic domain of RlmCD (Fig 2B and S2B Fig), confirming that RlmCD recognizes U747 site majorly through the loop region of 23S rRNA hairpin 35. In addition, two extra base pairs (^743^C-G^754^ and ^744^C-G^753^) in RlmCD-SAH-U747_SL_ show no obvious interaction with RlmCD, extending outward from the RNA catalytic groove. Given the higher resolution of RlmCD-SAH-U747_L_ complex structure, further analysis of protein-RNA interactions for U747-containing RNA substrates was mainly based on the RlmCD-SAH-U747_L_ ternary complex. Interestingly, in the RlmCD-SAH-U1939_L_ complex structure, the U1939-containing RNA substrate also adopts a very similar conformation to that of U747_L_, even though their sequences are totally different (Fig 2B and 2C), suggesting that both RNAs were re-shaped by the catalytic groove of RlmCD to adopt an optimal conformation for protein-RNA recognition and subsequent catalysis reaction. The nucleotide base-flipping was also observed for U1939 in the RlmCD-SAH-U1939_L_ structure (Fig 2B).

### Interaction details between RlmCD and U747_L_

**Fig 3:**
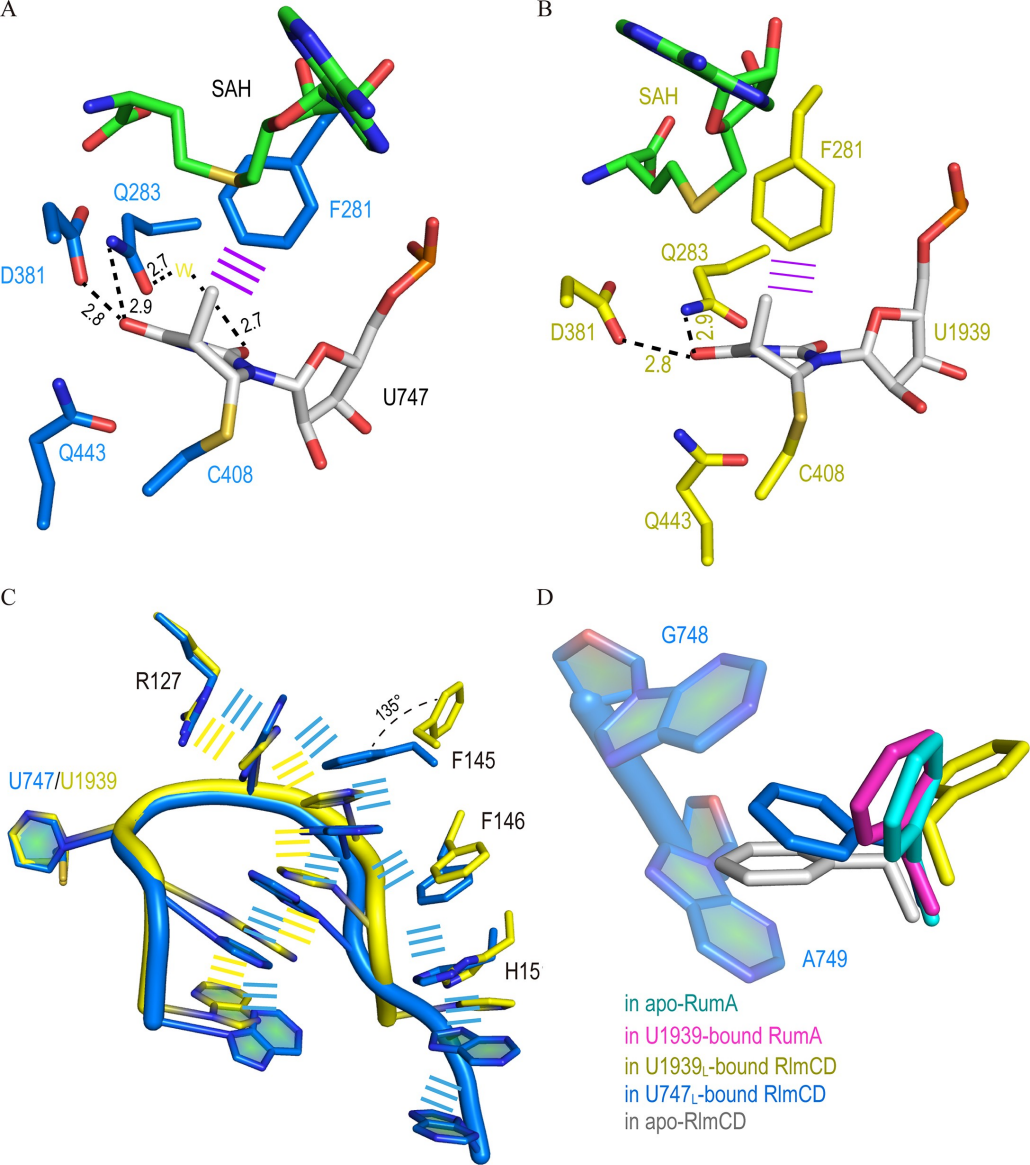
Protein-RNA recognition in complex structures of RlmCD-SAH-U747_L_ and RlmCD-SAH-U1939_L_. (A, B) Interaction details between nucleotide of U747 or U1939 and surrounding RlmCD residues. Hydrogen bonding interactions are all indicated as black dashed lines. The yellow “W” represents water molecule and the purple line indicates face-to-edge stacking. (C) Comparison of aromatic stacking interactions in RlmCD-SAH-U747_L_ (marine) and RlmCD-SAH-U1939_L_ (yellow). (D) Side-chain of F145 adopts multiple conformations in apo and RNA-bound structures of RlmCD, while its conformations remain similar in the apo or RNA-bound structure of RumA.

In the structure of RlmCD-SAH-U747_L_, the RNA substrate interacts with RlmCD significantly using a combination of hydrogen-bonding interactions and aromatic stacking. As the methylated target, the U747 base flips out of the RNA loop and interacts with residues within the active site of RlmCD. In detail, the U747 base forms multiple hydrogen bonds with the side-chain groups of D381, Q283, and Q443, while the O2′ and O3′ of U747 ribose contribute two hydrogen bonds with the side-chain of R384 (Fig 3A and S3A Fig). In addition, an edge-to-face aromatic interaction is formed between the U747 base and F281 side-chain. As the immediately adjacent nucleotide to U747, G748 also participates in the formation of two hydrogen bonds with the main chain of Q131 and the side-chain of Q162 using its base (S3A and S4A Fig). The above interactions relating to U747 and G748, plus a non-base specific hydrogen bond formed between the phosphate moiety of G748 and positively charged R127, contribute significantly to the RlmCD-U747_L_ interaction, ensuring that the protein and RNA substrate can recognize each other with high specificity. Moreover, non-base hydrogen bonds were also observed between the backbone phosphate groups of A749 and A750 and the side-chains of H441 and R127, as well as the backbone hydroxyl group of N149 (S4A and S4B Fig), while multiple hydrogen bonds were formed between the side-chains of N247 and N249 and the bases of G745, U746, A751 and A752 (S4B and S4C Fig), further stabilizing the U-shape conformation of U747_L_.

Another prevailing subject in the RlmCD-U747_L_ interaction is the π-π stacking between the nucleotide base and the aromatic ring or long side-chain of the protein residue. The aromatic ring of F145 inserts into the space between the bases of G748 and A749, pushing these two bases further away from each other and forming aromatic stacking with both. Intriguingly, the A749 base can further stack with A750 base and F146 aromatic ring simultaneously (S4D Fig). Consequently, two partially joined stacking networks (Arg127-G748-Phe145-A749-A750-U746-G745 and Arg127-G748-Phe145-A749-Phe146-His151-A751-A752) were formed as a “λ”-shape geometry in the complex structure of RlmCD-SAH-U747_L_. We proposed that, besides compensating the free energy cost of base flipping in the complex, this π-π stacking network may help to strengthen the recognition of RlmCD for the U747-containing RNA substrate.

### Interaction details between RlmCD and U1939_L_

Compared with U747, U1939 in the structure of RlmCD-SAH-U1939_L_ exhibits a totally identical interaction mode with RlmCD, suggesting that the catalytic mechanism RlmCD employed for two different RNA substrates are quite similar (Fig 3B and S3B Fig). As the corresponding nucleotide to G748 in U1939_L_, however, U1940 maintains only one hydrogen bond with the main chain hydroxyl group of Q131 using its base, while the Q131 main chain is also involved in two additional hydrogen bonds with the bases of C1941 and C1942, respectively (S4E Fig). N249, whose side-chain originally makes multiple hydrogen bonds with the bases of three different nucleotides in the RlmCD-SAH-U747_L_ structure, forms only one side-chain-to-base hydrogen bond with A1937 (S4F Fig). In addition, the side-chain groups of N249 and H151 make two alternative hydrogen bonds with the phosphate moiety of U1943 (S4G Fig). Similar hydrogen bonds were also observed between the phosphate groups of U1940 and C1941 and the side-chains of R127 and H441 (S4E Fig).

The most striking difference between the two complex structures of RlmCD is that the aromatic side-chain of F145 in RlmCD-SAH-U1939_L_ swings away from its position in RlmCD-SAH-U747_L_ by an angle of ~135°, without disturbing the base-stacking between U1940 and C1941 (Fig 3C). In addition, the side-chain of F146 also shifts by ~1.4 Å away from RNA nucleotide. As a result, only one base stacking network extended by R127 (Arg127-U1940-C1941-C1942-A1938-A1937-A1936) is formed in RlmCD-SAH-U1939_L_ (S4H Fig). Because much fewer protein residues are involved in the stacking interactions with RNA, one could expect that the existing base stacking interactions in RlmCD-SAH-U1939_L_ contribute significantly to the conformational stability of RNA rather than the protein-RNA recognition.

### RlmCD shares a similar fundamental catalytic mechanism with RumA

**Fig 4:**
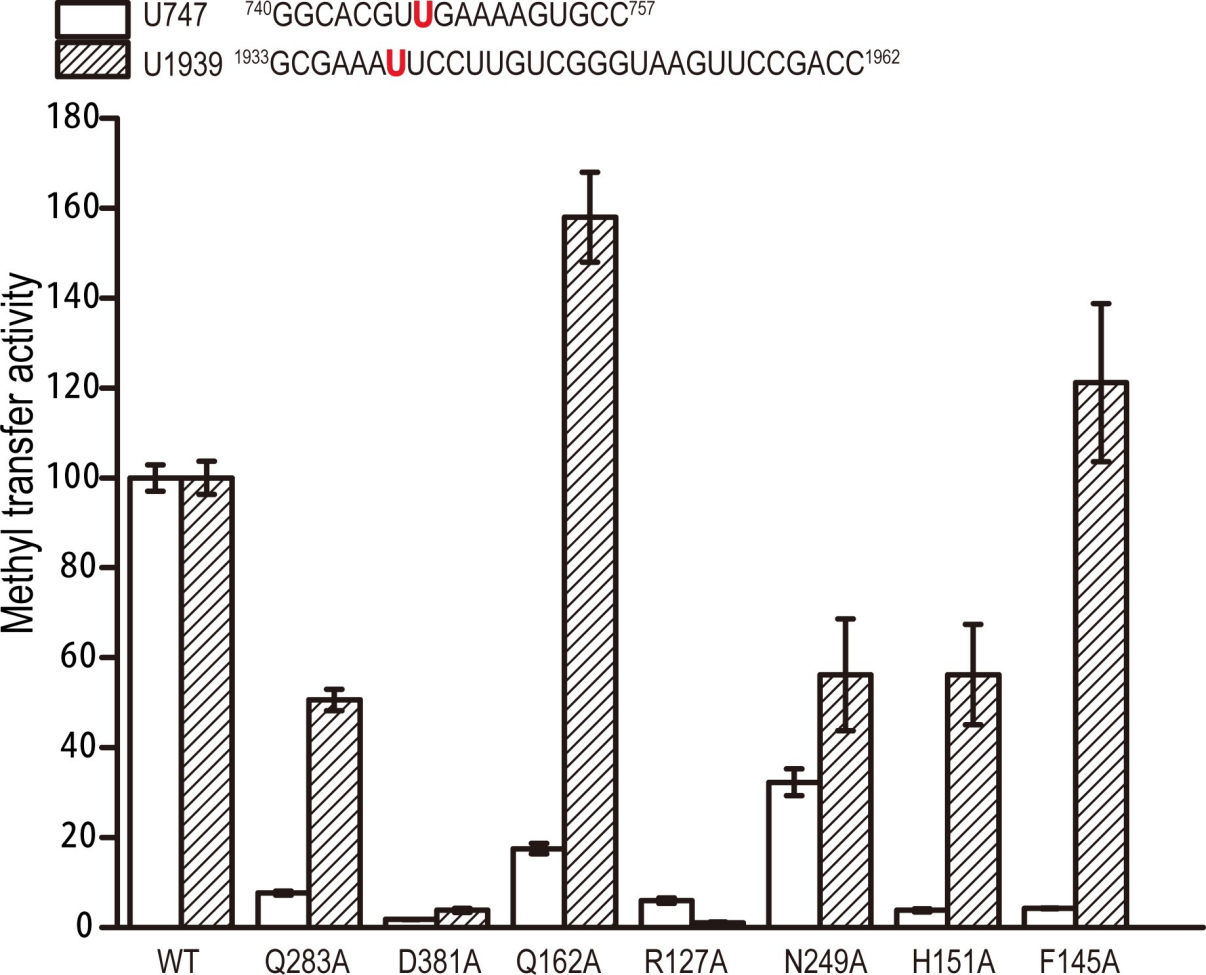
Crucial residues for MTase activities of RlmCD towards U747 and U1939 RNA substrates. Comparison of MTase activities of wild-type RlmCD and its mutants using an U747-containing RNA segment analogous to 23S rRNA hairpin 35 or U1939-containing RNA segment representing 1933–1962 of 23S rRNA as substrate. MTase activity of wild-type RlmCD was normalized to 100%. Error bars indicate the standard error of three separate measurements.

As described above, both the bases U747 and U1939 insert into the active site of RlmCD, interacting with exactly the same list of residues (F281, Q283, D381, and Q443) from the C-terminal catalytic domain by either a hydrogen bond or face-to-edge aromatic interaction. The first three residues and E443 are all highly conserved in RumA-like enzymes (S5 Fig). Because it was previously proven that E443 functions as the general base in RlmCD for the methylation reaction and F281 contributes significantly to RlmCD’s enzymatic activity [29], Q283A and D381A mutants were further generated for in-vitro MTase assay and electrophoretic mobility shift assay (EMSA) in this research. As summarized in Fig 4, both Q283A and D381A showed obvious experimental effects towards the U747 RNA substrate, retaining ~8% and ~2% of the MTase activity of wild-type RlmCD, while they exhibited ~50% and ~4% MTase activity of wild-type RlmCD towards the U1939 RNA substrate. In addition, our EMSA results showed that the RNA-binding capacities of Q283A and D381A are not severely affected by mutation compared with wild-type RlmCD (S6 and S7 Figs). Our experimental evidence indicated that both Q283 and D381, similarly as their equivalent residues in RumA (Q265 and D363), are necessary components of the active site of RlmCD for its specific recognition of uracil, and the hydrogen-bonding capacity retained by D381 appears more important than previously suggested [25].

### Q162 and N249 assist in establishing recognition specificity of RlmCD for U747 site of 23S rRNA

As the residues from the central domain of RlmCD, R127 forms a hydrogen bond with the backbone phosphate group of G748 or U1940, using its side-chain, and extends the cation-π stacking interaction at these nucleotides in both complex structures, while Q162 forms a side-chain-to-base hydrogen bond with G748 solely in RlmCD-SAH-U747_L_. The influence on the MTase activity and RNA binding of RlmCD by R127 and Q162 was therefore investigated by site-directed mutagenesis (Fig 4). Compared with the R127A mutant that abolishes the enzymatic activities of RlmCD against U747 and U1939 RNAs by ~92% and ~98%, respectively, Q162A retains ~18% MTase activity for U747 RNA but gains ~60% more MTase activity for U1939 RNA. However, both mutants exhibited obviously reduced RNA binding capacities compared with wild-type RlmCD towards the different RNA substrates (S6 and S7 Figs). These results suggested that R127 plays a pivotal role in consolidating the interaction of RlmCD with RNA substrate, while Q162 contributes to the MTase activity of RlmCD by offering recognition specificity rather than just binding affinity for the RNA substrate.

N249 is located within a linker region in the central domain of RlmCD, which is much longer than its counterpart in RumA [29]. In our complex structures, the N249 side-chain is involved in three hydrogen bonds with G745, U746, and A751 of U747_L_ while it forms two hydrogen bonds with A1937 and U1943 of U1939_L_. Correspondingly, N249A preserves ~35% and ~55% of the MTase activity of wild-type RlmCD towards U747 and U1939 RNA substrates (Fig 4), consistent with its EMSA results, in which the RlmCD binding abilities for U747 and U1939 RNAs both decrease, but with a more dramatic effect towards the U747 RNA (S6 and S7 Figs). Together, our experimental evidence suggested that N249 may also assist in establishing the recognition specificity of RlmCD for 23S rRNA hairpin 35.

### Side-chain rearrangement of F145 is a delicate mechanism that allows RlmCD to switch between its two catalytic capacities

As described above, in the RlmCD-SAH-U747_L_ structure, several aromatic residues of RlmCD, including F145, F146, and H151, stack with U747_L_ nucleotides, forming extensive stacking interactions between the protein and RNA. In the RlmCD-SAH-U1939_L_ structure, however, the aromatic side-chain of F145 rotates by ~135° so that it remains distant from the bases of U1940 and C1941, isolating the aromatic residues of RlmCD from RNA base stacking (Fig 3C). To testify whether this stacking is important for the RlmCD-U747_L_ interaction, site-directed mutants were applied for the MTase assay and EMSA (Fig 4 and S6 and S7 Figs). As expected, both F145A and H151A mutants retain ~5% of the MTase activity of the wild-type RlmCD for U747 RNA substrate. In the case of the U1939 RNA substrate, however, H151A only causes a ~45% reduction in MTase activity, whereas F145A results in no significant effect (~20% increase). On the other hand, the RNA binding capacities of H151A for U747 and U1939 RNAs both fell moderately, as suggested by the EMSA results, while F145A causes a minor reduction in RlmCD binding for U747 RNA but almost no obvious effect for U1939 RNA. The above results confirmed that H151 contributes to the protein-RNA recognition as an important participant in the intermolecular stacking in RlmCD-SAH-U747_L_ but becomes less important in RlmCD-SAH-U1939_L_ due to the destruction of protein-RNA stacking interactions, even though it still interacts with 3′ end of U1939_L_ through a non-base specific hydrogen bond. Although it is not likely that F145 shows significant impact on RlmCD binding for 23S rRNA, the distinctive effect of F145A on the MTase activities of RlmCD for U747 and U1939 RNAs suggested that, while RumA is a dedicated enzyme for m^5^U1939, side-chain rearrangement of F145 appears to be a delicate mechanism used by RlmCD to fine tune its binding and/or recognition for RNA substrates so that RlmCD can switch between its two catalytic abilities.

### Binding mode comparison between RlmCD-SAH-U1939_L_ and RumA-SAH-RNA

**Fig 5:**
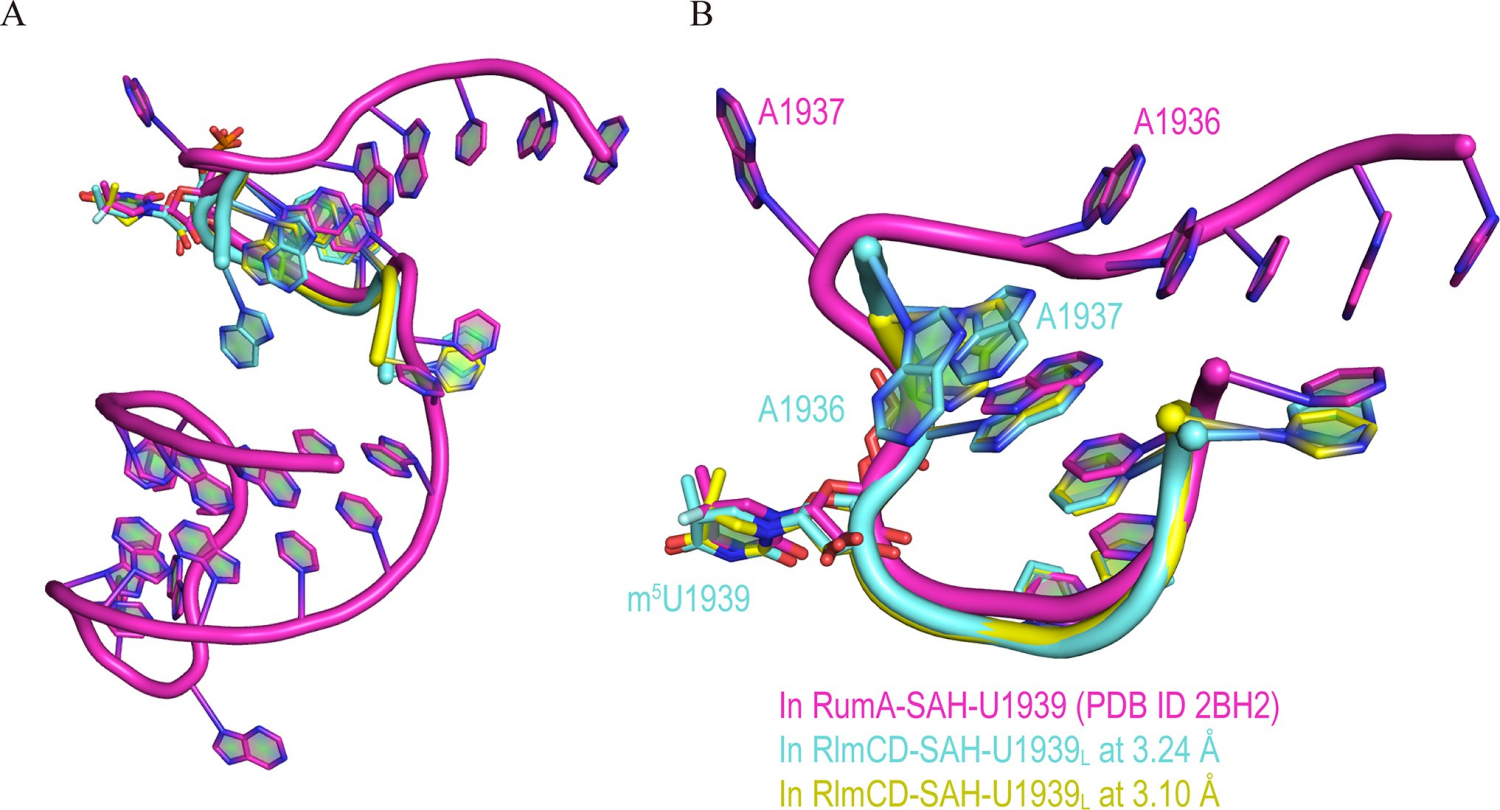
RNA-recognition mode of RlmCD may differ from that of RumA. (A) Conformational superimposition of U1939_L_ from 3.10 Å (yellow) and 3.24 Å (aquamarine) RlmCD-SAH-U1939_L_ structures (PDB ID 5ZQ1 and 5ZTH) and 30-nt U1939-containing RNA (1932–1961) (magenta) from RumA-SAN-RNA structure (PDB ID 2BH2) [25]. (B) Close-up of conformational superimposition of corresponding region in two RNAs.

Lee et al. previously investigated the RNA recognition of RumA for a 30-nt RNA segment representing A1932-C1961 of *E*. *coli* 23S rRNA using X-ray crystallography. In this structure, the 5′-end region (A1932-U1943) binds at the catalytic groove formed by the central and catalytic domains of RumA, while the 3′-side hairpin (G1945-C1961) binds in the cleft formed between the N-terminal OB fold domain and the catalytic domain [25]. Because the RlmCD-SAH-U1939_L_ structure we determined in this research contains only the A1936-U1943 of 5′-end loop, we therefore focused on a comparison of the RNA binding within the catalytic grooves of RumA and RlmCD. The majority of nucleotides within A1936-U1943 maintain a very similar conformation in the two complex structures, except A1936 and A1937 (Fig 5). In the RumA-SAH-RNA structure, A1937 is flipped out of the loop to form an edge-to-face stacking interaction with the adenine ring of SAH, shielding the cofactor from solvent; this is a function usually conducted by protein residues in many other SAM-MTases [25, 32, 33]. The energy cost of A1937 flipping is balanced by Van der Waals interaction between the A1938 base and A1936 ribose, which abruptly bends the orientation of the RNA backbone at A1937. In the RlmCD-SAH-U1939_L_ structure, however, no base flipping of A1937 was observed, and A1937 forms base stacking with adjacent A1938 when the electron density of A1936 is incompletely observed. In fact, in an alternative RlmCD-SAH-U1939_L_ structure we determined at 3.24 Å resolution (Table 1), the electron density of A1936 is clear and three sequential adenines (A1938, A1937, and A1936) form three layers of base stacking, leading the RNA backbone towards an orientation distinct from that in the RumA-SAH-RNA structure (Fig 5 and S8 Fig). As the consecutive basic patch on the surface of the central domain of RumA where A1932-G1935 binds is largely eliminated by two extra linkers (linker A and B) in RlmCD (Fig 1A) [29], it may not be surprising that RNA adjusts its backbone orientation for the 5′-side nucleotides of A1936 so that they can bind RlmCD more appropriately. Overall, our structural evidence indicated that RlmCD maintains a similar but not identical recognition mode for U1939-containing RNA substrate to that of RumA, which may be related to the modified RNA-binding capacity of the central domain in RlmCD.

## Discussion

### Multiple factors contribute to dual enzymatic activities of RlmCD

In this research, we determined the crystal structures of RlmCD in complex with two RNA segments containing U747 and U1939. Careful analysis of protein-RNA recognition between the catalytic groove of RlmCD and these two RNA segments uncovered several crucial residues (F145, Q162 and N249) that are responsible for its dual enzymatic activity towards C5 methylation of U747 and U1939, especially when these residues are all strictly conserved in YefA (S5 Fig).

Of these residues, N249 behaves differently in interactions of RlmCD and two different RNA substrates but with a more significant impact on U747 methylation, confirming our previous conclusion that the linker containing N249 is a newly evolved structural determinant that helps RlmCD to obtain additional MTase activity for U747 [29]. As another important residue in discriminating RNA substrates, Q162 is not conserved in RlmC and RumA (S5 Fig). Its equivalent residue L165 in RumA remains distant from U1940 and exhibits no interaction with the RNA in the complex structure of RumA-SAH-RNA [25], similarly as Q162 in RlmCD-SAH-U1939_L_. Considering the opposite effects of Q162A mutation on MTase activities of RlmCD for U747 and U1939 RNAs, we concluded that although Q162 may not seem to be an evolutionary mutation beneficial for RlmCD MTase activity towards U1939, it does establish the binding specificity of RlmCD for U747-containing RNA.

F145, on the other hand, is a common residue in RlmCD, YefA (F152), and RumA (F148). Unlike the RumA structures in which the conformations of F148 side-chain remain almost the same with and without RNA substrates, the structures of RlmCD in the absence and presence of U747_L_ and U1939_L_ show that F145 adopts multiple conformations for its side-chain under different circumstances (Fig 3D). Upon binding with U747_L_, F145 side-chain slightly changes its orientation from that in the apo-form structure so that it can stack with G748 and A749 properly. In the complex structure of RlmCD-SAH-U1939_L_, however, F145 largely re-orients its side-chain and remains distant from RNA nucleotides. We observed that through aromatic side-chain re-orientation, F145 switches between two distinctive conformational states that are optimized for the recognition of U747 and U1939 RNAs.

Overall, our crystal structures of RlmCD complexed with U747_L_ and U1939_L_ unveil multiple structural features to explain how RlmCD can specifically recognize U747 RNA while it remains enzymatically functional for the methylation of U1939. The aromatic side-chain rearrangement of F145 was observed to be an unusual strategy used by RlmCD to discriminate between two different sites on 23S rRNA by switching on/off the intermolecular π-π stacking between protein and RNA; dit Konté and coworkers recently reported a similar mechanism employed by RRM3 (RNA recognition motif 3) of CUG-BP2 (GUG triplet repeats RNA binding protein) to discriminate its two U-rich RNA substrates [34]. Both this work and our research implicate that aromatic residue(s) that is/are located on the protein-RNA binding interface may sometimes be the crucial factor determining whether a protein can recognize multiple RNA substrates.

### Evolutionary implication of dual enzymatic activities of RlmCD for 23S rRNA

It has been suggested that all contemporary m^5^U RNA MTases evolved from a common ancestor, which is most likely an RumA-like enzyme that originated amongst bacteria [35]. RumA homologues have been found to exist in various bacteria and are responsible for the C5 methylation of U1939, while two other *E*. *coli* m^5^U MTase, RlmC and TrmA [31], are restricted to Proteobacteria, and their catalytic targets are shifted to U747 of 23S rRNA and U54 in tRNA, respectively [36]. In recent years, RlmCD and YefA were shown to have dual enzymatic activities towards both U747 and U1939 in some gram-positive bacteria, showing a typical example of divergent evolution in the rRNA methylation machinery [37].

Although both RlmCD and RlmC can introduce C5 methylation at U747 of 23S rRNA, their RNA-recognition mechanisms may differ, as the residues contributing to the enzymatic activity of RlmCD for U747 (F145, Q162, and N249) are not conserved in RlmC, and RlmC does not possess a TRAM domain (S5 Fig). Most likely, RlmCD acquired extra MTase activity for U747 via gene duplication during a later evolutionary process from an RumA-like origin rather than inheriting this feature from a RlmC-type enzyme through horizontal gene transfer. From an evolutionary perspective, the acquisition of new function might represent a branch node on the phylogenetic tree. Are the dual enzymatic activities of RlmCD for different m^5^U sites on 23S rRNA unique to *B*. *subtilis* and *S*. *pneumoniae*, or is this feature prevalent in gram-positive bacteria? Based on our structural findings in this research, a further bioinformatics study should be employed to provide hints to answer this question.

### Single-site mutant of F145 can assist in functional exploration of U747 methylation

*S*. *pneumoniae* is the major cause of bacterial pneumonia and meningitis, as well as bloodstream, ear, and sinus infections. Due to the global antibiotic resistance crisis, it has been estimated that ~1.2 million illnesses and ~7,000 deaths per year are caused by *S*. *pneumoniae* infection. Its drug resistance has been listed as one of serious threats to human health by the CDC [38]. In 23S rRNA, m^5^U747 locates in the loop region of hairpin 35, protruding into the large subunit tunnel for extension of the nascent peptide. Although U747 methylation has been recently found to contribute to TEL susceptibility through promoting efficient RlmA^II^-mediated G748 methylation in *S*. *pneumoniae*, its precise functional role must be further illustrated. The m^5^U747 is close to NPET and itself may become a target for the design of new antibiotics against diseases and syndromes induced by *S*. *pneumoniae*.

In this research, although multiple residues were shown to contribute simultaneously to the specific recognitions of RlmCD for two different sites on 23S rRNA, the single mutant F145A alone can abolish the MTase activity of RlmCD for U747 while its enzymatic activity for U1939 remains almost intact, exhibiting a relatively ‘clean’ effect in decoupling the dual MTase activities of RlmCD; however, this result must be further confirmed by in-vivo experiments. F145A therefore provides us with an unprecedented opportunity to illustrate the functional role of U747 methylation in cells without affecting the methylation level of U1939 in 23S rRNA under normal conditions and certain stress conditions, which may eventually assist in the development of new antimicrobial drugs targeting at m^5^U747 in *S*. *pneumoniae* or other related pathogens [39, 40].

## Material and methods

### Cloning, expression and purification

The RlmCD (sp_1029) ORF was amplified from *S*. *pneumoniae* genomic DNA via PCR. The full-length RlmCD (residue 1–543) and C-terminal truncated RlmCD (residue 1–454) were subcloned into a modified pET28a (Novagen) vector with an 8×His-SUMO tag and a ULP1 cleavage site (pET-SUMO vector) at the N terminal site. All single residue mutants were generated using MutanBEST kit (TaRaKa) and verified by DNA sequencing. The plasmids were transformed into *E*. *coli* BL21-Gold (DE3) cells.

For wild-type and mutant protein expression, cells were grown in LB medium at 37°C until the OD_600_ reached 0.8, and isopropyl β-D-1-thiogalactopyranoside (IPTG) was added to a final concentration of 0.2 mM. After induction, cells were grown at 16°C for an additional 24 hours before harvesting. The cell pellets were suspended with binding buffer (20 mM Tris-HCl, 2 M NaCl, pH 8.0), and lysed by sonication. After centrifugation, the supernatant was purified using Ni-NTA affinity chromatography (Qiagen). The eluted SUMO-tag protein was loaded on Superdex 200(16/60) (GE healthcare) equilibrated with binding buffer for further purification. The elution sample was mixed with ULP1 enzyme against storage buffer (20 mM Tris-HCl, 250 mM NaCl, pH 8.0) to remove the SUMO tag. After overnight cleavage, the mixture was further purified by Superdex 200(16/60) (GE healthcare), again with storage buffer.

### RNA preparation

Unmodified and 5′-6-carboxyfluorescein (FAM)-labelled RNA oligomers, including U747_L_ RNA (5′-^745^GUUGAAAA^752^-3′), U747_SL_ RNA (5′-^743^CCGUUGAAAAGG^754^-3′), FAM-U747_SL_ RNA (5′-FAM-^743^CCGUUGAAAAGG^754^-3′), U1939_L_ RNA (5′-^1935^AAAUUCCU^1943^-3′), 30-nt FAM-U1939 RNA (5′-FAM-^1933^GCGAAAUUCCUUGUCGGGUAAGUUCCGACC^1962^-3′) were purchased from TaRaKa Bio Inc. and dissolved in diethyl pyrocarbonate (DEPC)-treated water to a final concentration of 2 mM. The 30-nt U1939 RNA (5′-^1933^GCGAAAUUCCUUGUCGGGUAAGUUCCGACC^1962^-3′) for the in-vitro MTase assay was obtained using in-vitro RNA transcription by T7 RNA polymerase, as previously described [29]. The nucleotide at 1933 in 30-nt U1939 RNA was mutated to G from original C for a more efficient transcription and the same RNA sequence was maintained in 30-nt 5′-FAM-labelled U1939 RNA oligomer for consistency. All the RNA samples were denatured by heating to 98°C for 5 min followed by subsequent slow cooling to room temperature (25°C) for refolding.

### Crystallization

RlmCD (1–454) E443Q mutant was concentrated to ~6 mg/mL in storage buffer (20 mM Tris-HCl, 250 mM NaCl, pH 8.0). RlmCD concentration was determined by its ultraviolet absorption at wavelength 280 nm. The extinction coefficient of full-length RlmCD was 0.78 and RlmCD (1–454) was 0.71. The enzyme-cofactor-RNA covalent complex was prepared by incubating 0.117 mM RlmCD with 0.234 mM SAM and the 0.129 mM RNA substrate at a molecular ratio of 1:2:1.1 in storage buffer supplemented with 1 mM MgCl_2_ and 10% DEPC at 298 K for 1 hour.

The initial crystallization trial was set up using the hanging drop vapor diffusion method at 293 K. Each sample was mixed with buffer in a 1:1 ratio to equilibrate against a 100-μL reservoir solution. The crystals started to appear after 1 week at 293 K. Crystals of RlmCD-SAH-747_L_ for data collection were finally refined under 0.1 M HEPES, pH 7.5, 36%(w/v) PEG600; Crystals of RlmCD-SAH-747_SL_ were finally refined under 0.2 M ammonium acetate, 0.1 M sodium acetate, pH5.2 20% (w/v) PEG3350; Crystals of RlmCD-SAH-1939_L_ were finally refined under 0.1 M MES, pH 5.5, 0.15 M ammonium sulfate, 25% PEG4000. All the pH was adjusted at 298K.

### Data collection, structure determination and model refinement

The size suitable crystals (fitted with 0.1–0.2 mm cryoloop) were transferred directly into cryo-protectant flash-frozen into liquid nitrogen. The cryo-protectant was prepared by mixing 7 μL crystallization buffer with 3 μL glycerol. X-ray diffraction data were collected at the Shanghai Synchrotron Radiation Facility (SSRF) Beamline 19U1 or 17U. Intensity data were indexed and scaled by HKL2000. All complex structures were solved by molecular replacement with the program MOLREP in CCP4i using apo RlmCD (PDB ID: 5XJ1) as the search model [41]. The RNA models were built manually with COOT under electron density map contoured at 1.0σ. All structure refinements were performed using REFMAC5 and COOT interchangeably [42]. PyMOL (DeLano Scientific) was used to prepare the structure figures and calculate the RMSD values for Cα atoms.

### Electrophoretic mobility shift assay (EMSA)

EMSA was performed in a 10-μL reaction mixture containing 20 mM Tris-HCl, pH 8.0, 250 mM NaCl, and 1 mM MgCl_2_. The final concentrations of FAM-labelled U747_SL_ RNA or 30-nt U1939 RNA and SAH were 0.5 and 5μM. The final concentrations of RlmCD or its mutants are 0, 0.1, 0.2, 0.4, 0.8, 1.0, 2.5 and 5.0 μM, as indicated in S6 and S7 Figs. Reaction mixtures were incubated at 37°C for 30 minute before loading onto a 10% non-denaturing polyacrylamide gel and then electrophoresed on ice for 40 minutes at a constant voltage of 120 V. The 10% non-denaturing polyacrylamide gel was prepared by mixing 3.9 mL ddH_2_O, 1.5 mL 40% Acrylamide: Bis-acrylamide (19:1), 0.6 mL 10X TBE buffer, 30 μL 10% APS, and 3 μL TEMED. Subsequently, gels were scanned using a Typhoo FLA 7000 biomolecular imager.

### In-vitro methyltransferase assay

The MTase assay was performed as previously described [29].

## Supporting information

S1 Fig Close-up of active site in RlmCD-SAH-U747_SL_ (A) and RlmCD-SAH-U1939_L_ (B). Electron density maps with 2Fo-Fc calculated at 1.0σ shown for SAH, RlmCD C408, and all RNA nucleotides. (C) The proposed catalytic mechanism of m^5^U MTase RlmCD.
(TIF)

S2 Fig (A) In crystal of RlmCD-SAH-U747_SL_ complex, each asymmetry unit contains two complex molecules (lime and pink) that pack together through RNA-RNA intermolecular base-stacking. (B) Superimposition of complex structures of RlmCD-SAH-U747_L_ (marine) and RlmCD-SAH-U747_SL_ (pink) with an RMSD for Cα atoms of 0.4 Å. (*Inset*) Close-up of overall structures of U747_L_ and U747_SL_. Orientation of structures is slightly adjusted for clarity.
(TIF)

S3 Fig Schematic view of protein-RNA interactions in RlmCD-SAH-U747_L_ (A) and RlmCD-SAH-U1939_L_ (B).
(TIF)

S4 Fig (A-C) Interaction details between individual nucleotides of U747_L_ (except U747) and surrounding RlmCD residues. (D) Aromatic stacking in RlmCD-SAH-U747_L_ structure. RlmCD residues in different complex structures are shown in blue and yellow, respectively. (E-G) Interaction details between individual nucleotides of U1939_L_ (except U1939) and surrounding RlmCD residues. (H) Aromatic stacking in RlmCD-SAH-U1939_L_ structure.
(TIF)

S5 Fig Sequence alignment of full-length *Streptococcus pneumoniae* RlmCD, *Bacillus subtilis* YefA (Sequence Identity 34.7%), *Escherichia coli* RumA (Sequence Identity 18.8%) and *Escherichia coli* RlmC (Sequence Identity 16.6%) are calculated by Clustal Omega. Conserved residues are shown in white on a red background, and similar residues are shown in red in a blue rectangle. Conserved residues participating in U747 recognition between RlmCD and YefA (except for those from catalytic domain) are labeled with a black asterisk[43].
(TIF)

S6 Fig Electrophoretic mobility shift assay of wild-type RlmCD and its mutants using 5′-FAM-labelled U747_SL_ RNA as substrate. Free RNA and shifted protein-RNA complex are labelled.
(TIF)

S7 Fig Electrophoretic mobility shift assay of wild-type RlmCD and its mutants using 5′-FAM-labelled 30-nt U1939-containing RNA (1933–1962) as substrate. Free RNA and shifted protein-RNA complexes are labelled. Two shifted bands for protein-RNA complexes are observed in all EMSA experiments of U1939 RNA and we proposed that the upper band may be induced by the nonspecific binding of 30-nt RNA for an extra RlmCD molecule under high protein concentrations.
(TIF)

S8 Fig (A) Superimposition of 3.10 Å (yellow) and 3.24 Å (aquamarine) RlmCD-SAH-U1939L complex structures. (*Inset*) Close-up of overall structures of U1939L RNAs in two models. (B) Stick model of U1939L RNA in 3.24 Å RlmCD-SAH-U1939L complex structure. 2Fo-Fc electron density map is calculated at 1.0σ.
(TIF)
