# Supplementary figures and images for: Unveiling the structural features that determine the dual methyltransferase activities of Streptococcus pneumoniae RlmCD
Source: PLoS Pathog. 2018 Nov 2;14(11):e1007379. doi: 10.1371/journal.ppat.1007379 (PMC6235398; doi:10.1371/journal.ppat.1007379)

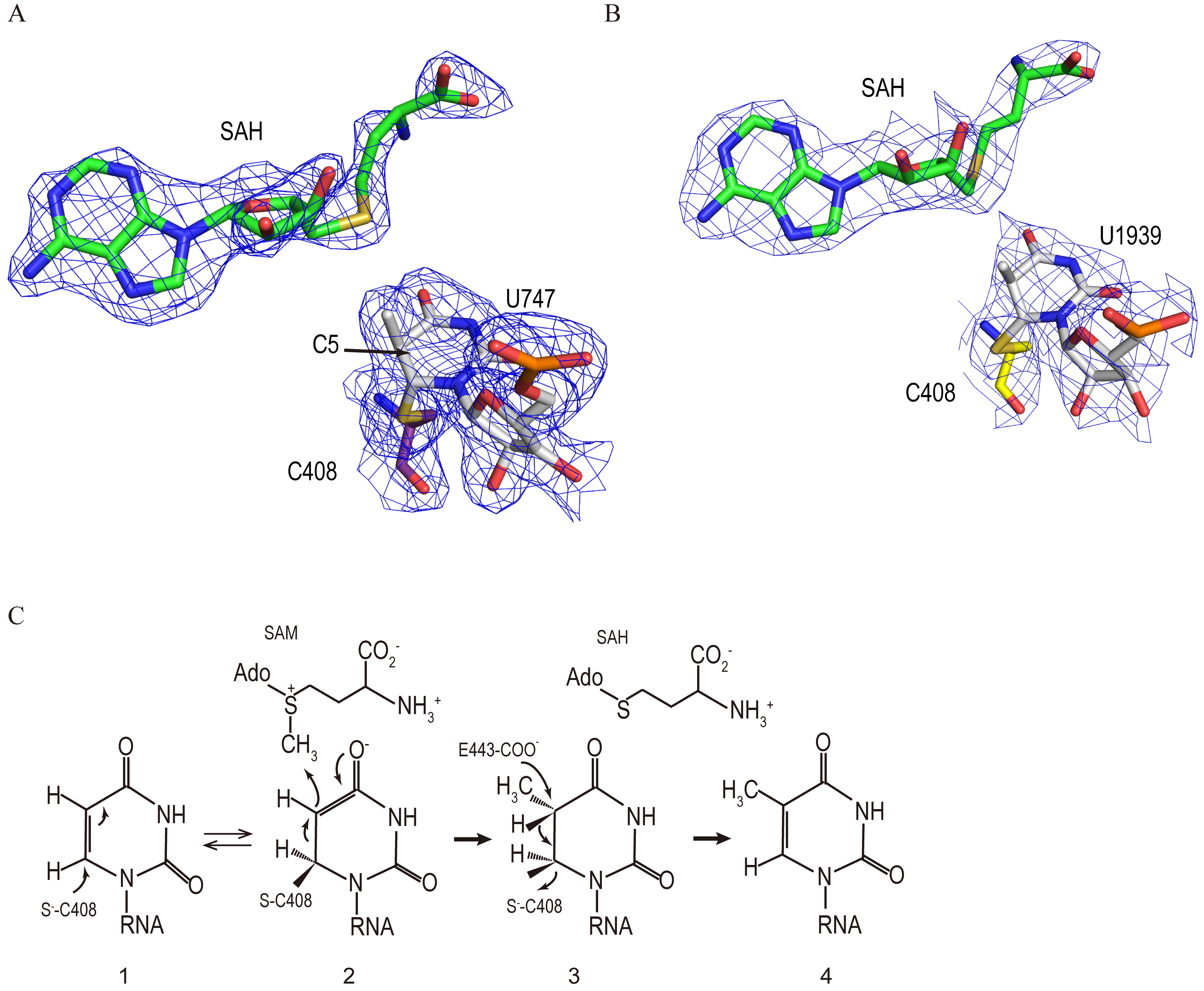

Supplement: S1 Fig — Close-up of active site in RlmCD-SAH-U747SL (A) and RlmCD-SAH-U1939L (B). Electron density maps with 2Fo-Fc calculated at 1.0σ shown for SAH, RlmCD C408, and all RNA nucleotides. (C) The proposed catalytic mechanism of m5U MTase RlmCD. (TIF) [file ppat.1007379.s001.tif]

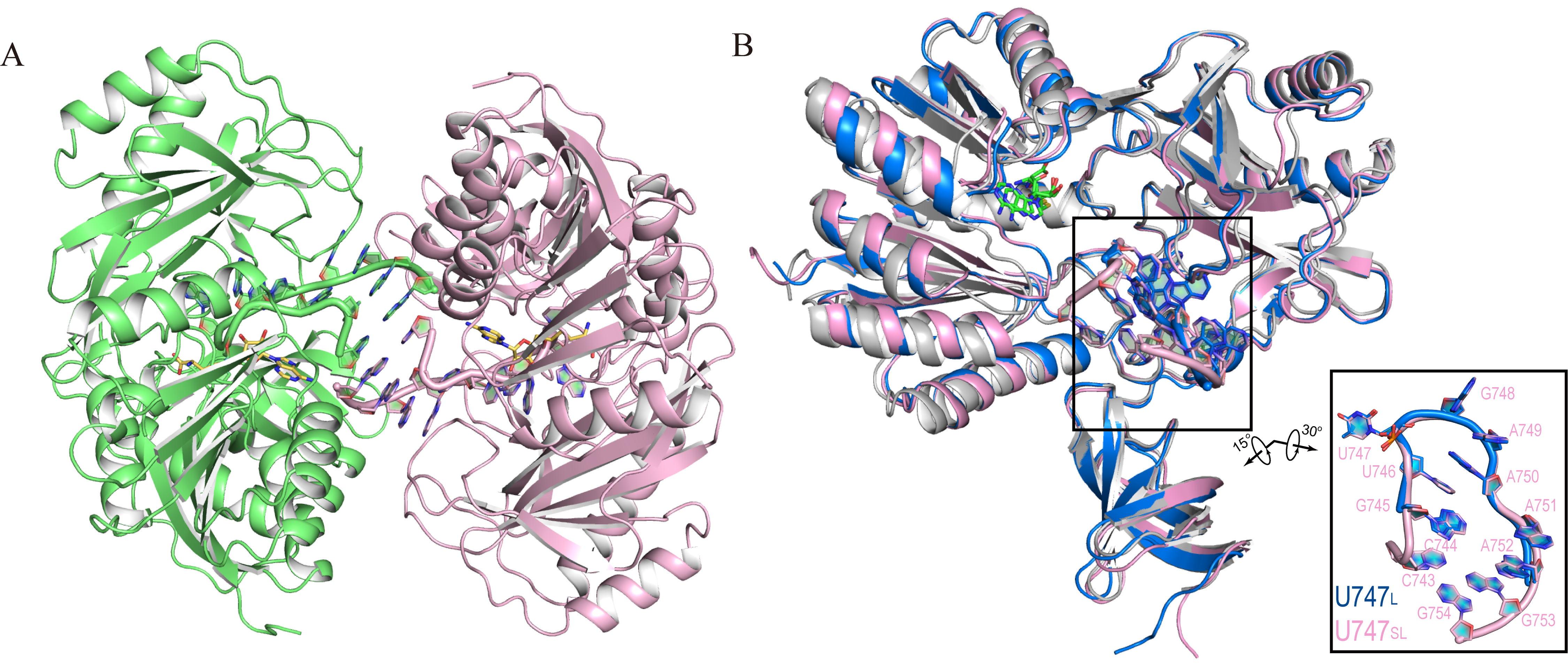

Supplement: S2 Fig — (A) In crystal of RlmCD-SAH-U747SL complex, each asymmetry unit contains two complex molecules (lime and pink) that pack together through RNA-RNA intermolecular base-stacking. (B) Superimposition of complex structures of RlmCD-SAH-U747L (marine) and RlmCD-SAH-U747SL (pink) with an RMSD for Cα atoms of 0.4 Å. (Inset) Close-up of overall structures of U747L and U747SL. Orientation of structures is slightly adjusted for clarity. (TIF) [file ppat.1007379.s002.tif]

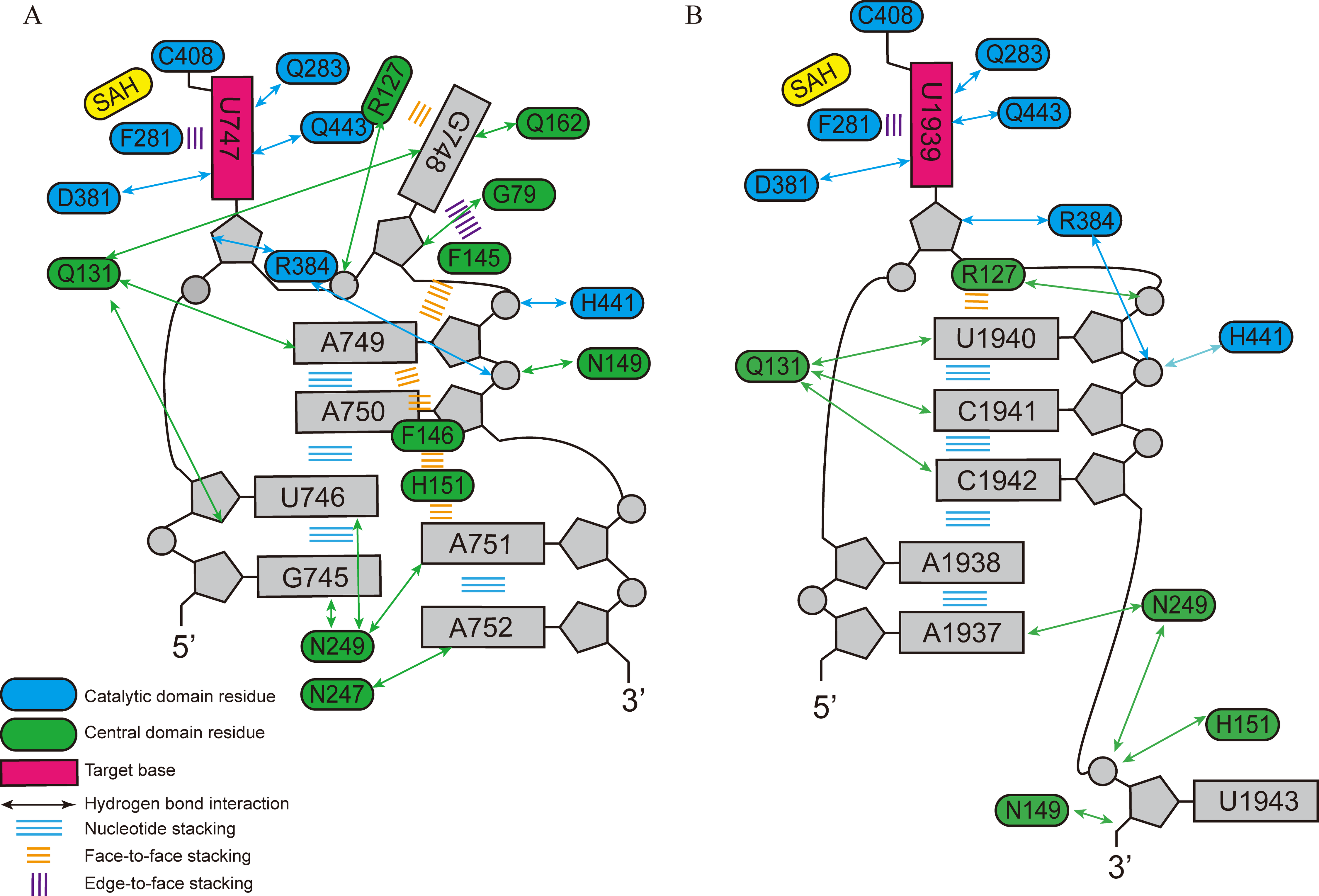

Supplement: S3 Fig — Schematic view of protein-RNA interactions in RlmCD-SAH-U747L (A) and RlmCD-SAH-U1939L (B). (TIF) [file ppat.1007379.s003.tif]

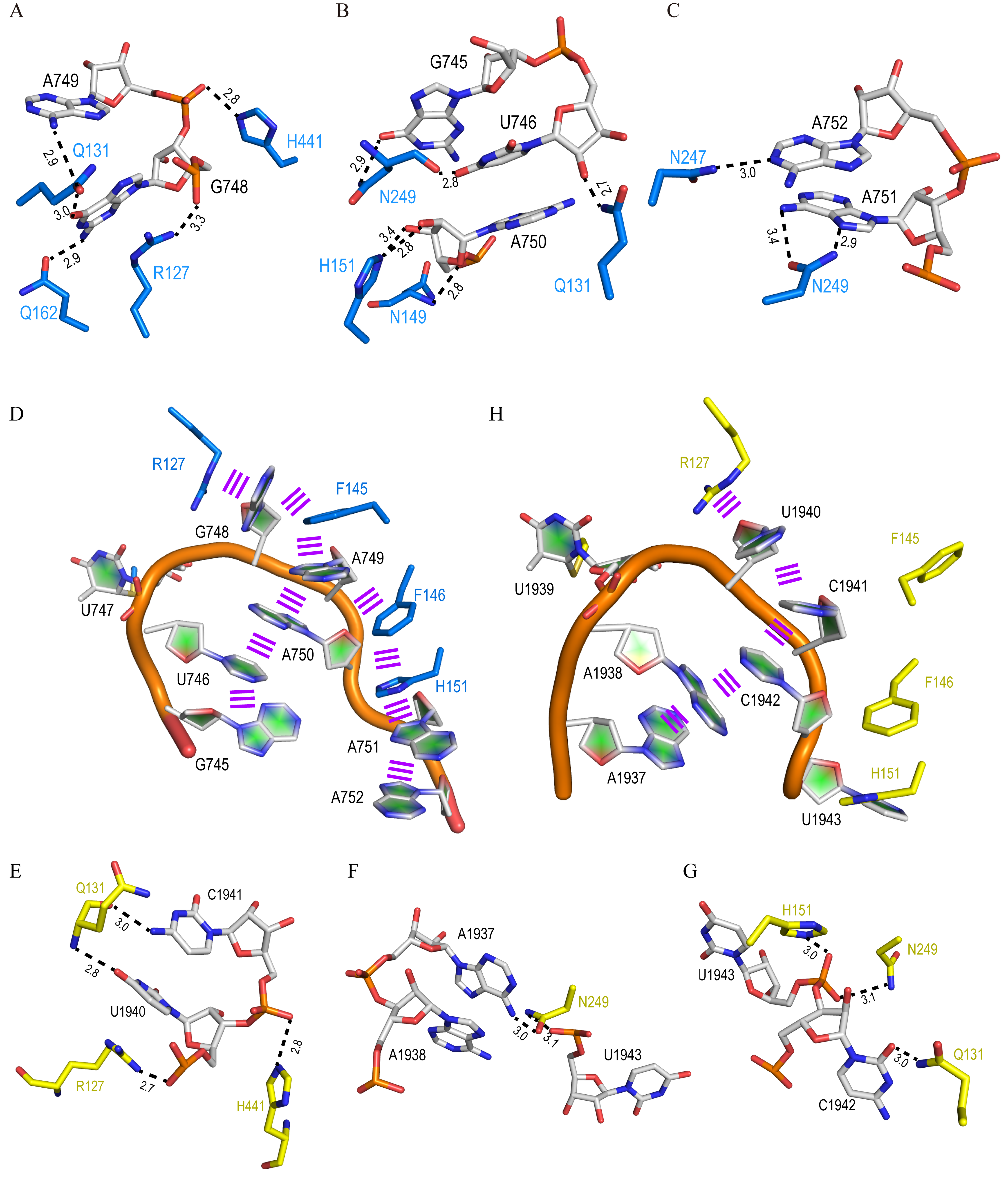

Supplement: S4 Fig — (A-C) Interaction details between individual nucleotides of U747L (except U747) and surrounding RlmCD residues. (D) Aromatic stacking in RlmCD-SAH-U747L structure. RlmCD residues in different complex structures are shown in blue and yellow, respectively. (E-G) Interaction details between individual nucleotides of U1939L (except U1939) and surrounding RlmCD residues. (H) Aromatic stacking in RlmCD-SAH-U1939L structure. (TIF) [file ppat.1007379.s004.tif]

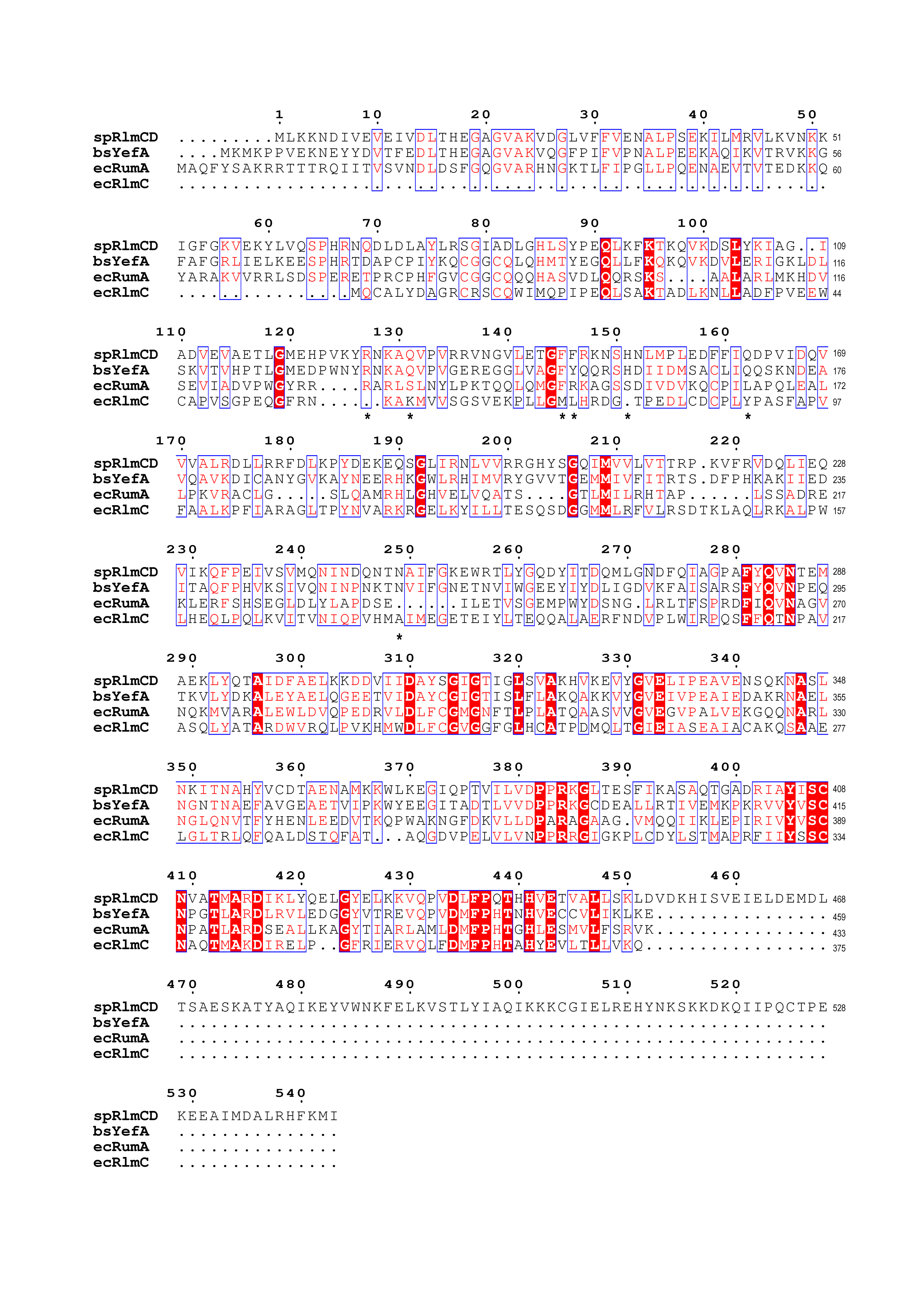

Supplement: S5 Fig — Conserved residues are shown in white on a red background, and similar residues are shown in red in a blue rectangle. Conserved residues participating in U747 recognition between RlmCD and YefA (except for those from catalytic domain) are labeled with a black asterisk[43]. (TIF) [file ppat.1007379.s005.tif]

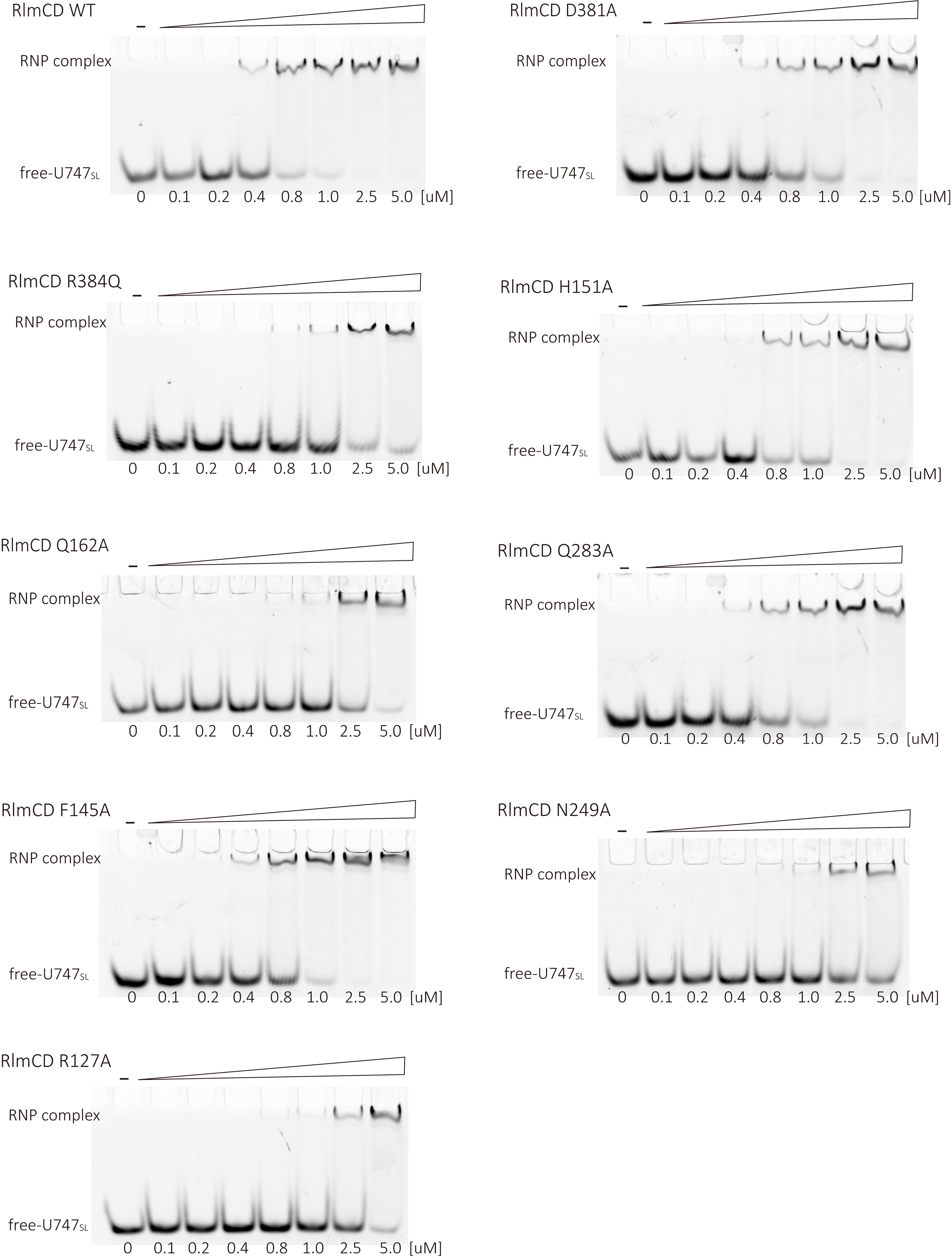

Supplement: S6 Fig — Free RNA and shifted protein-RNA complex are labelled. (TIF) [file ppat.1007379.s006.tif]

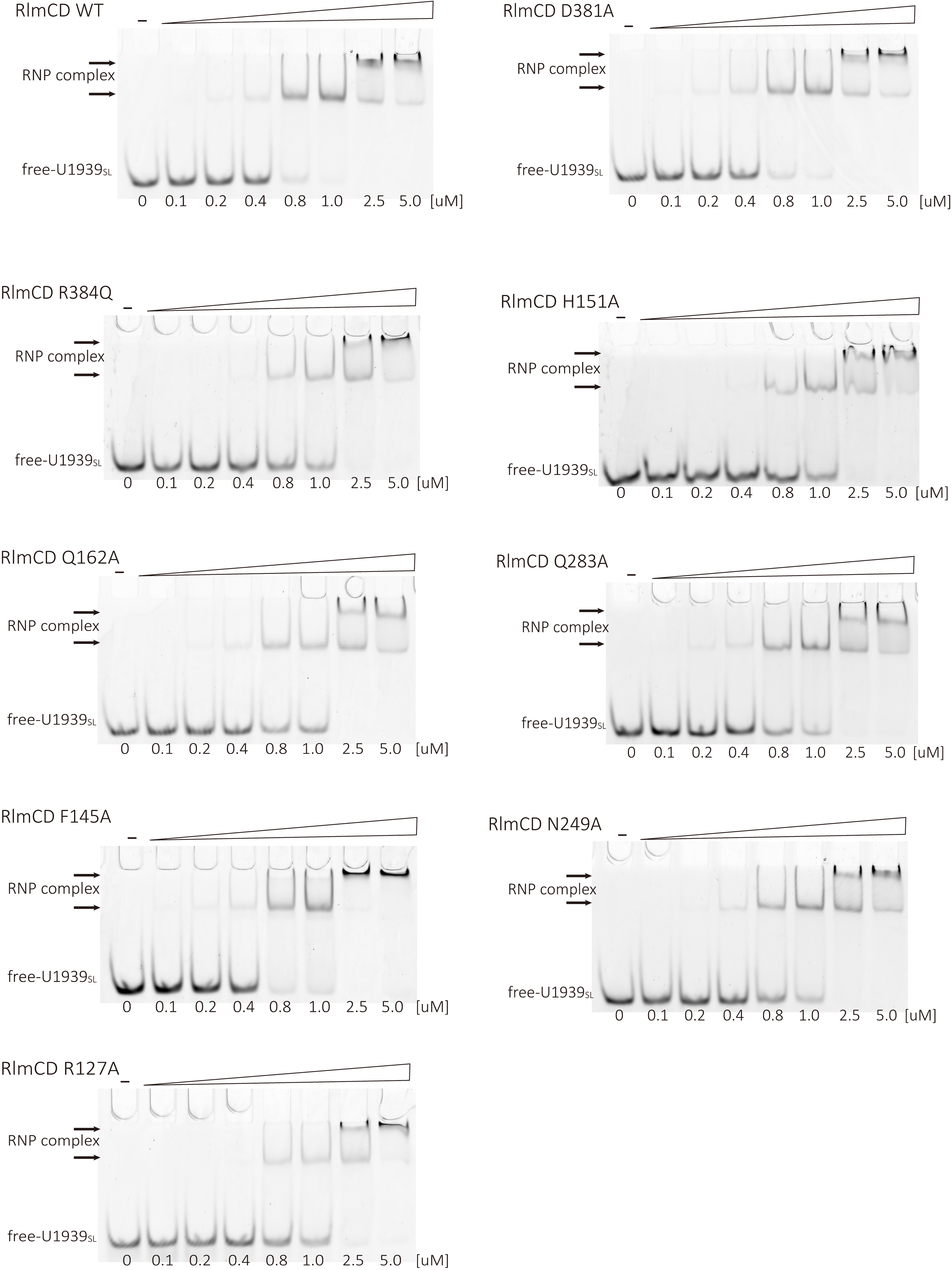

Supplement: S7 Fig — Free RNA and shifted protein-RNA complexes are labelled. Two shifted bands for protein-RNA complexes are observed in all EMSA experiments of U1939 RNA and we proposed that the upper band may be induced by the nonspecific binding of 30-nt RNA for an extra RlmCD molecule under high protein concentrations. (TIF) [file ppat.1007379.s007.tif]

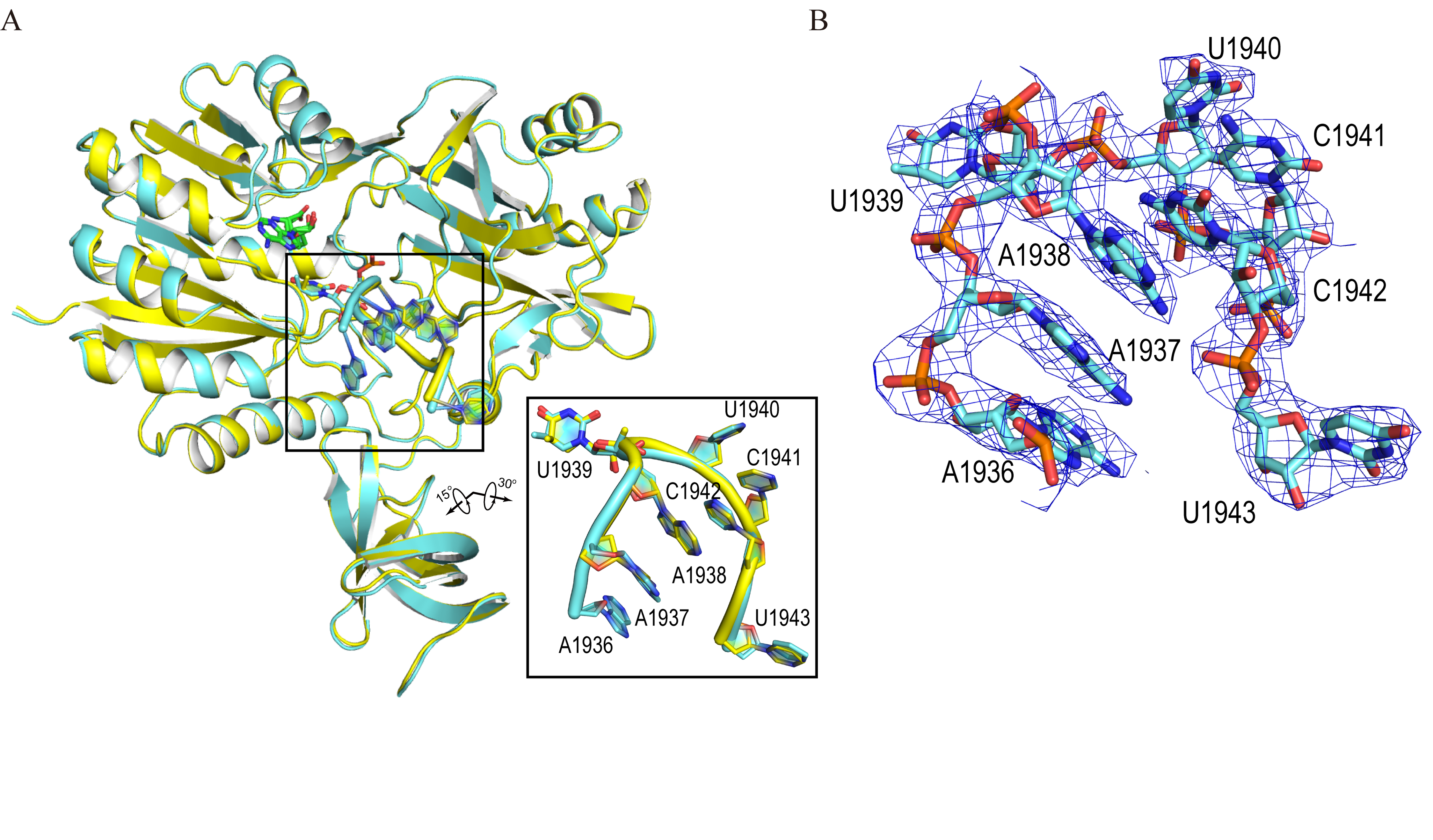

Supplement: S8 Fig — (A) Superimposition of 3.10 Å (yellow) and 3.24 Å (aquamarine) RlmCD-SAH-U1939L complex structures. (Inset) Close-up of overall structures of U1939L RNAs in two models. (B) Stick model of U1939L RNA in 3.24 Å RlmCD-SAH-U1939L complex structure. 2Fo-Fc electron density map is calculated at 1.0σ. (TIF) [file ppat.1007379.s008.tif]
